# The Covid-19 Pandemic and Maternal Mental Health: A Longitudinal Study of Chilean and Foreign-Born Mothers

**DOI:** 10.3389/ijph.2022.1604724

**Published:** 2022-07-13

**Authors:** Alejandra Abufhele, Marigen Narea, Amanda Telias

**Affiliations:** ^1^ Center for Advanced Studies on Educational Justice, Santiago, Chile; ^2^ School of Psychology, Pontificia Universidad Católica de Chile, Santiago, Chile

**Keywords:** COVID-19, longitudinal study, maternal mental health, parental stress, Chile

## Abstract

**Objectives:** We explore the effects of the pandemic on stress, depressive symptoms and parenting practices of mothers with children aged between 24- and 30-months, residents in Santiago, Chile, and the differences between foreign‐born and native‐born mothers.

**Methods:** Using data from the longitudinal project *Mil Primeros Días* and lagged-dependent models, we analyzed parental stress, depressive symptoms and parenting practices for native-born and foreign-born mothers. Lagged-dependent model allows us to take advantage of the longitudinal data by controlling for the previous score and baseline individual characteristics.

**Results:** After 8 months of the pandemic, mothers of young children have more depressive symptoms, are more stressed, and show more hostility towards their children. Foreign-born mothers had 0.29 and 0.22 standard deviations (SD) more than native-born mothers in the parental distress and difficult child scales from the Parental Stress Index (PSI), respectively, and 0.17 SD more in the hostile-reactive parental behavior dimension.

**Conclusion:** Findings suggest the need to implement policies and programs that prevent mental health deterioration for mothers, especially migrant mothers, to improve women’s psychological condition and child wellness.

## Introduction

The COVID-19 world pandemic has affected the mental health of individuals not only because of the illness and widespread loss of life but also because most countries have implemented lockdowns for long periods, together with other restrictive measures that have limited mobility and reduced available resources. A group that has been particularly affected is parents, especially mothers [1], who have confronted additional demands, such as working from home, homeschooling, economic difficulties, and social restrictions [2, 3]. Several studies have tried to measure the impacts of the COVID-19 pandemic on mental health. Early findings showed that mental health issues are arising in many countries. A study for the UK found that mental health has deteriorated compared with pre-pandemic trends, and more considerable impacts were associated with particular populations like young adults (18–34 years) and people living with young children [4]. Another study from Bangladesh examined different outcomes and presented evidence that mothers’ depression and anxiety symptoms have increased during lockdown [5].

Of particular relevance is to study pandemic effects on parenting practices and mothers’ mental health with young children. It has been demonstrated that early childhood is a crucial stage for later development and avoiding intergenerational handovers of mental health problems [6]. It has been shown that maternal depression is associated with children with lower cognitive performance and academic achievement, more significant behavioral problems, demanding temperaments, and poor physical growth in young children in developing countries [7, 8].

Evidence about the pandemic’s harmful effects on maternal mental health is still scarce, especially for mothers with younger children. Evidence from developed countries has found substantial adverse impacts on the mental health of parents with children [9–12]. A recent study for India [13] found that parents reported moderate to high perceived stress since the COVID-19 lockdown, and they reported feeling more stressed in their role as parents. There is only one study for Latin America focused on pandemic effects on mental health in vulnerable mothers. For Tumaco, Colombia, a conflict-affected municipality, a study [14] found a considerable deterioration in anxiety, depression, and parenting stress associated with COVID-19. On average, post-pandemic cohorts presented a 14-percentage point higher probability of reporting anxiety; a 5-percentage point increased probability of depression, and a 10-percentage point higher probability of parental stress.

Our study focuses on the effects of the pandemic on stress, depressive symptoms and parental practices, for mothers of young children. In addition, and considering that south-south migration has been prolific during the last decade, and Chile has been one of the critical recipient countries, we explore if this effect differs depending on native-born and foreign-born mothers. The recent immigration wave in Chile has no parallel in its history. While in 2006, 1.0% of the population was born outside of Chile (150 thousand immigrants), in 2017, this percentage was 4.4% (800 thousand immigrants), and preliminary estimates for 2018 placed immigrants at more than 1 million. Moreover, almost half of the migrant population are women who arrived during childbearing ages [14]. It has been shown that immigration status can be a disadvantageous situation in particular contexts, potentially intensifying the effects of the pandemic on mental health and having consequences for well-being far beyond the pandemic [14]. In Chile, migrant mothers can be considered vulnerable for two reasons. First, some migrants do not have their legal paperwork updated, so they are not eligible or cannot access the government social protection system. Second, compared to Chilean mothers, migrant mothers reported not having a supportive care system during the pandemic, they lost their jobs, and a substantial proportion of them experienced a change of household during the pandemic [15].

We use longitudinal pre-pandemic data and post-pandemic data from 8 months after the pandemic start from 985 families, concentrating on mothers with children between 24 and 30 months of age. Our sample has 18.4% migrant mothers, i.e., foreign-born mothers. The first data collection was in 2019, while the second round was in 2020 after mandatory lockdowns were implemented in the country. We aimed to analyze how the COVID-19 pandemic has affected foreign-born and native-born mothers. This paper contributes to the literature by assessing the effect of the COVID-19 pandemic on maternal mental health and parental practices - stress, depressive symptoms and hostility towards infants—in Chile, focusing on mothers of small children and exploring two subgroup populations: native-born and foreign-born mothers. Studying the effects of the pandemic on mental health is crucial to prevent further consequences that mental health issues could have and take action that can mitigate these effects.

## Methods

### Data

**FIGURE 1:**
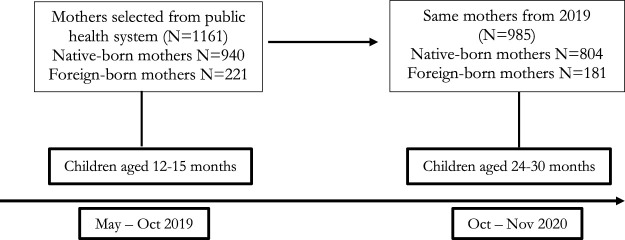
Sample, number of observations and ages of children (Mil Primeros Días, Santiago, Chile, 2019–2020).

The data used in this study comes from the Chilean Longitudinal Study *Mil Primeros Días* (One Thousand First Days), which is a two-round study conducted by the Center for Advanced Studies on Educational Justice (CJE) of the Pontificia Universidad Católica de Chile [16]. The first round was implemented in 2019, while the second data collection was between August and November 2020. The sample used includes the same mothers surveyed in the two rounds (*n* = 985) who had children between 24 and 30 months in the second round. See Figure 1.

The sample provides information from native-born and foreign-born families from different socioeconomic backgrounds. The sample of mothers was selected from users of the public health system in Santiago. The invitation to enroll in the longitudinal study was made to mothers who took their infant to their 12-month-old health check at the public primary health care. Public health system users represent approximately 80% of the population in Chile, and the other 20% are treated in the private health system. The first wave was a face-to-face survey that gathered two types of information: a socio-demographic survey and tests for evaluating cognitive, language, and socio-emotional development in children and their mothers. Due to the COVID-19 pandemic, the second wave was implemented by a phone survey.

Sociodemographic variables of our study’s sample coincide with the comparison group in the Chilean National Socioeconomic Characterization Survey (CASEN). For example, the median household income in our sample matches the median income of the first four quintiles in CASEN. The richest income quintile in CASEN is not represented in our sample [15].

Of the total participants of the first wave it was possible to apply the telephone survey to 84.8%, which corresponds to 985 cases. There was 10.6% of the sample that was not able to contact, 4% that didn’t answer the phone and only 1% refused to answer. In the second wave, 81.6% of the sample corresponds to Chilean mothers and 18.4% to foreign-born mothers, compared to the 81.0% of Chilean and 19.0% of foreign-born mothers in the first wave [15].

### Measures

We used three different instruments to measure maternal depressive symptoms, stress, and parental practices. The Center for Epidemiological Studies-Depression (CES-D), Parent Stress Index Scale (PSI), and Parental Cognitions and Conduct Towards the Infant Scale (PACOTIS). The Center for Epidemiological Studies-Depression [17] is a 20-question instrument aimed at detecting cases of depression based on the presence of symptoms associated with depression that have occurred during the week before the application of the test. A total score is obtained from all the responses, and a high score implies higher depressive symptoms. In the first wave, we applied the CES-D-20 to a subsample and an abbreviated 10-item version to the other subsample. We used Item Response Theory (IRT) to score both instruments (CES-D-20 and the 10-item version) on a standard scale and compare their properties. We adopted the 10-item version for the data collection’s second wave based on this procedure. This instrument has been used in Chile to measure the prevalence of depression in mothers of young children [18, 19].

The second instrument is the Parent Stress Index Scale (PSI) Short Form, a self-applied instrument that helps identify sources and types of stress that arise in those who become parents. It is divided into three sub-scales: Parental distress (PD), Dysfunctional parent-child interaction (P-CDI), and Difficult child (DC). The PD subscale refers to how parents feel competent, restricted, conflicted, supported, or depressed in their role as a parent. The P-CDI scale refers to the extent to which parents feel satisfied with their child and their interactions with them, and the DC scale measures how a parent perceives their child to be, whether the child is easy or difficult to take care of [20]. Based on the analysis of the dataset from the first round and following the study of other academic research [21–23], a 15-item reduced version was generated through the use of factorial analyses techniques and was applied in the second wave to facilitate its telephone application [15]. This instrument has already been used in Chile [21].

The last instrument is the Parental Cognitions and Conduct Towards the Infant Scale (PACOTIS) [24]. This self-applied scale measures perceptions and parental behavior with a newborn child. It has four dimensions: parental self-efficacy, hostile-reactive parental behavior, parental hostility, and overprotection. In this study, we only use the hostile-reactive parental behavior dimension. This scale has seven items. An example of the questions asked is: “When my child cries, he/she makes me nervous” or “I have hit my child when he/she has been particularly fussy.”

For all instruments, an Item Response Theory (IRT) score was created and normalized to a z-score (M = 0 and SD = 1) [19]. For each test, the interpretation of the scores is a higher value; it means higher depressive symptoms, higher stress levels, and higher hostility. For CES-D (20 items) in wave one and the CES-D adapted version in wave two (10 items), the partial credit model (PCM) weighted mean likelihood (WLE) estimation reliability coefficient was 0.74, which is considered a reasonable IRT reliability estimate [19]. For PSI-SF (36 items) and the adapted version (15 items), the reliability coefficients were 0.75 for the Parental Distress subscale, 0.71 for the Parent-Child Dysfunctional Interaction subscale, and 0.75 for the Difficult Child subscale [19]. For our key coefficients to be more meaningful, we standardized the IRT scores concerning the IRT scores in wave one. Hence, all standardized scores have an average equal to zero and a standard deviation equal to one in wave one [19].

### Statistical Analysis

The statistical analysis included two sets of estimations using a lagged-dependent model, which allows us to take advantage of the longitudinal data by controlling for the previous score and baseline individual characteristics. First, we estimated linear regression models for the depressive symptoms, parenting, and stress scale with the whole sample using an independent variable identifying if the mother is a migrant or not. Secondly, we calculated the same linear regressions but one for foreign‐born mothers and another for native‐born mothers.

In the first set of models, the main independent variable is binary, which indicates if the mother is foreign-born or native-born. For the second set of models, we separated the estimations by groups to account for possible heterogeneities between the native and foreign-born. In all the analyses, we controlled for the lagged value of each test to consider basal levels of maternal stress, depressive symptoms, and parenting practices. Maternal and household characteristics were included as covariates. Variables associated with the mother include years of schooling, age, binary variables, whether she was working in 2019 or not, and whether she was married. Household variables include a categorical variable of overcrowding, income per capita, and a standardized IRT score of the instrument Home Observation for Measurement of the Environment Inventory (HOME), which measures the quality of home environments and interactions. Its purpose is to measure the quality and quantity of stimulation and support available to the child in their home [25]. We also include a binary variable that identifies when the family had a housing change during the period in between waves.

Regarding the context of the COVID-19 pandemic, we used a set of covariates potentially associated with mental health—first, a variable of the days of mandatory lockdown for the borough. Also, we include a categorical variable that indicates if the person completed lockdown fully, partially, or failed to meet. Regarding economic matters, binary variables were included showing whether the household reduced its income due to COVID-19 or not, whether it received financial help from the government, and whether it withdrew money from its retirement fund. Finally, we included two binary variables that account for other special shocks: a family member having COVID-19 during the period or not and if they had a family member that died from COVID-19 in the period or not to account for possible grief.

## Results

Table 1 (panel A) shows the difference in the five outcomes—depressive symptoms, parental distress, difficult child, child dysfunction interaction, and hostility—before and after the onset of the pandemic. After 8 months of the pandemic, which included prolonged lockdowns, mothers of young children have more depressive symptoms, are more stressed, and show more hostility towards their children. Mothers are more stressed in two out of three dimensions of the PSI (DC and CDI). The post covid means are higher in these four outcomes than the pre covid means, and all of these differences are statistically significantly using a mean comparison test (*t*-test).

**TABLE 1:** Maternal Depressive symptoms, Stress and Hostility—2019 (Pre COVID-19 pandemic) and 2020 (COVID-19 pandemic) (Mil Primeros Días, Santiago, Chile, 2019–2020).

Panel A: All	2019				2020				Mean comparison test T-Test		Obs.
	Pre COVID-19 pandemic				COVID-19 pandemic						
	Mean	SD	Min	Max	Mean	SD	Min	Max	t	Sig	
CES-D IRT	0.000	1.000	−3.530	2.911	0.189	1.121	−2.641	3.176	−3.939	***	985
PSI-PD IRT	0.000	1.000	−3.955	4.744	−0.366	1.21	−3.197	4.063	7.307	***	985
PSI-DC IRT	0.000	1.000	−3.221	3.026	0.626	0.999	−2.496	5.038	−13.9	***	985
PSI-CDI IRT	0.000	1.000	−1.625	3.216	0.296	1.021	−1.218	2.190	−6.509	***	985
PACOTIS - Hostility IRT	0.000	1.000	−2.184	3.626	0.18	0.846	−2.184	2.704	−4.303	***	981
Panel B: Native-born Mothers	Mean	SD	Min	Max	Mean	SD	Mean	SD	t	Sig	
CES-D IRT	0.032	1.013	−3.530	2.911	0.204	1.131	−2.641	3.176	−3.196	**	804
PSI-PD IRT	0.016	0.982	−3.955	4.744	−0.396	1.222	−3.197	4.063	7.449	***	804
PSI-DC IRT	−0.006	1.012	−3.221	3.026	0.63	0.997	−2.496	5.038	−12.688	***	804
PSI-CDI IRT	0.000	0.998	−1.625	3.216	0.257	1.030	−1.218	1.982	−5.114	***	804
PACOTIS—Hostility IRT	0.007	0.992	−2.184	3.626	0.149	0.845	−2.184	2.704	−3.095	***	801
Panel C: Foreign-born Mothers											
CES-D IRT	−0.146	0.929	−3.530	2.416	0.12	1.073	−2.641	2.845	−2.518	**	181
PSI-PD IRT	−0.07	1.074	−3.955	1.967	−0.229	1.148	−3.197	2.138	1.361		181
PSI-DC IRT	0.028	0.946	−3.221	1.950	0.610	1.010	−2.496	5.038	−5.665	***	181
PSI-CDI IRT	0.001	1.052	−1.625	1.657	0.471	0.968	−1.218	2.190	−4.419	***	181
PACOTIS—Hostility IRT	−0.031	1.038	−2.184	2.490	0.315	0.841	−2.184	2.704	−3.478	***	180

Panel B and C from Table 1 show the pre-and post covid means and standard deviations of the five outcomes for native-born mothers and foreign-born mothers, respectively. The differences pre- and post-covid reveal that native-born mothers show an increase of 0.17 on average on the depressive symptoms scale, while foreign-born mothers show an increase of 0.27. Regarding the parental stress scale, foreign-born mothers also show a higher increase in the child dysfunctional interaction dimension than native-born mothers. However, the native-born mothers present a higher increase after 8 months of the pandemic in terms of the difficult child dimension. The foreign-born mothers show an increase in the hostile dimension of PACOTIS twice that of the native-born growth.

Table 2 shows for all of the samples and each group—native-born and foreign-born mothers—descriptive statistics for all the covariates from the analytical sample. Besides some maternal and household characteristics, our data shows a gap favoring native-born mothers in government help received due to COVID-19 and their retirement money withdrawal. In contrast, more foreign-born mothers had a house change than the native-born group of mothers. The differences align with the evidence that households with foreign-born mothers can be exposed to more precarity and less access to government benefits.

**TABLE 2:** Descriptive statistics by native and foreign-born mothers (Mil Primeros Días, Santiago, Chile, 2019–2020).

	All sample	Native-born mothers	Foreign-born mothers
	Mean—%	Mean—%	Mean—%
Years of schooling	12.7	12.5	13.4
Age	29.6	29.4	30.1
Income per capita (log)	11.47	11.4	11.6
Home IRT Std	0.01	0.1	−0.4
Married [ref. not married]	63%	59%	81%
Working [ref. not working]	32%	31%	34%
Overcrowding			
No overcrowding	83%	88%	62%
Medium overcrowding	10%	8%	21%
High overcrowding	4%	3%	11%
Critical overcrowding	2%	2%	6%
Days of lockdown	126.2	125.7	128.4
Lockdown			
Failed to complete lockdown	8%	8%	7%
Partially complete lockdown	27%	27%	24%
Fully complete lockdown	66%	65%	69%
Income decrease due to Covid [ref. no decrease]	68%	67%	73%
Received help due to Covid [ref. no help]	91%	92%	86%
Withdraw of retirement money [ref. no retirement]	84%	87%	69%
Change of housing [ref. no change]	20%	18%	28%
Family member with Covid [ref. no Covid]	66%	67%	60%
Grief due to covid [ref. no grief]	24%	24%	26%
Number Observations	828	679	149

We estimated a lagged-dependent model for each mental health outcome, controlling for the standardized score for the same outcomes obtained in wave one and considering the main independent variable indicating whether the mother is foreign-born or not (binary variable). Results from this model are presented in Table 3. Secondly, we run the same model but separate the estimations into two groups, one for native-born mothers and the other for foreign-born mothers, to explore heterogeneities between the groups in the determinants of depressive symptoms, stress, and maternal hostility after 8 months of the pandemic. Table 4 presents the results for native-born mothers and Table 5 for foreign-born mothers.

**TABLE 3:** Linear regression model. All sample (Mil Primeros Días, Santiago, Chile, 2019–2020).

	CES-D	PSI-PD	PSI-DC	PSI-CDI	PACOTIS
	β	β	β	β	β
	CI 95%	CI 95%	CI 95%	CI 95%	CI 95%
Foreign-born Mother [ref. native-born mother]	0.063	0.288***	−0.087	0.217**	0.165*
	−0.144, 0.270	0.074, 0.501	−0.285, 0.110	0.029, 0.406	−0.001, 0.331
Years of schooling	−0.022	−0.017	−0.018	−0.016	−0.024**
	−0.051, 0.006	−0.047, 0.014	−0.042, 0.007	−0.044, 0.012	−0.045, −0.004
Age	0.003	0.019***	−0.003	0.005	0.012***
	−0.009, 0.016	0.007, 0.031	−0.013, 0.008	−0.006, 0.017	0.003, 0.021
Working [ref. not working]	−0.051	0.034	0.007	0.071	0.035
	−0.210, 0.108	−0.143, 0.210	−0.144, 0.159	−0.075, 0.218	−0.084, 0.153
Medium [ref. no overcrowding]	0.048	0.08	−0.019	−0.006	−0.106
	−0.186, 0.283	−0.158, 0.319	−0.209, 0.171	−0.236, 0.224	−0.278, 0.066
High [ref. no overcrowding]	−0.124	0.402**	−0.072	0.270*	−0.192
	−0.455, 0.207	0.050, 0.753	−0.348, 0.204	−0.034, 0.575	−0.433, 0.050
Critical [ref. no overcrowding]	−0.119	0.113	−0.158	−0.111	−0.236
	−0.510, 0.271	−0.380, 0.607	−0.645, 0.330	−0.573, 0.351	−0.664, 0.192
Married [ref. not married]	−0.058	−0.178**	−0.025	−0.221***	−0.032
	−0.206, 0.090	−0.338, −0.018	−0.159, 0.109	−0.366, −0.076	−0.146, 0.083
Income per−capita (log)	0.011	0.000	−0.026	0.003	−0.014
	−0.028, 0.050	−0.029, 0.029	−0.066, 0.013	−0.033, 0.040	−0.038, 0.009
Home IRT Std	0.045	0.011	−0.105***	−0.049	−0.019
	−0.033, 0.123	−0.070, 0.092	−0.177, −0.034	−0.124, 0.027	−0.086, 0.048
Days of lockdown	0.002	0.002	−0.002	0.001	0.001
	−0.000, 0.005	−0.001, 0.005	−0.004, 0.000	−0.002, 0.004	−0.001, 0.003
Partially complete lockdown [ref. failed to complete]	0.217	0.368**	0.067	0.249*	0.026
	−0.073, 0.507	0.024, 0.713	−0.200, 0.335	−0.026, 0.525	−0.167, 0.218
Fully complete lockdown [ref. failed to complete]	0.273**	0.377**	0.084	0.257*	−0.088
	0.001, 0.544	0.056, 0.698	−0.165, 0.333	−0.001, 0.515	−0.260, 0.085
Income decrease due to Covid [ref. no decrease]	−0.015	0.101	0.021	0.037	0.015
	−0.166, 0.137	−0.067, 0.270	−0.130, 0.172	−0.113, 0.186	−0.100, 0.130
Received help due to Covid [ref. no help]	0.102	0.260*	−0.051	0.215*	0.01
	−0.147, 0.351	−0.009, 0.529	−0.284, 0.182	−0.017, 0.447	−0.172, 0.192
Withdraw of retirement money [ref. no retirement]	0.105	0.274**	−0.003	0.213**	0.025
	−0.094, 0.303	0.042, 0.506	−0.183, 0.176	0.027, 0.400	−0.123, 0.174
Change of housing [ref. no change]	0.076	0.132	0.227***	0.163*	0.115*
	−0.111, 0.264	−0.061, 0.326	0.073, 0.382	−0.006, 0.332	−0.013, 0.243
Family member with Covid [ref. no Covid]	0.096	0.003	0.113	0.031	0.094
	−0.056, 0.248	−0.161, 0.166	−0.024, 0.250	−0.113, 0.174	−0.022, 0.210
Grief due to covid [ref. no grief]	0.176**	−0.012	−0.033	−0.027	−0.066
	0.010, 0.343	−0.197, 0.173	−0.195, 0.129	−0.189, 0.134	−0.196, 0.063
CES-D IRT Std W1	0.440***				
	0.366, 0.515				
PSI-PD IRT Std W1		0.445***			
		0.364, 0.526			
PSI-DC IRT Std W1			0.307***		
			0.234, 0.380		
PSI-CDI IRT Std W1				0.219***	
				0.149, 0.289	
PACOTIS IRT Std W1					0.348***
					0.285, 0.411
Constant	−0.519	−1.848***	1.337***	−0.458	0.167
	−1.344, 0.307	−2.665, −1.032	0.624, 2.050	−1.257, 0.341	−0.384, 0.718
Observations	828	828	828	828	828
R−squared	0.186	0.190	0.141	0.097	0.209

***p < 0.01, **p < 0.05, *p < 0.1.

Omitted/reference categories are mentioned following the ref—abbreviation term.

**TABLE 4:** Linear regression models native-born mothers (Mil Primeros Días, Santiago, Chile, 2019–2020).

	CES-D	PSI-PD	PSI-DC	PSI-CDI	PACOTIS
	Β	Β	β	β	β
	CI 95%	CI 95%	CI 95%	CI 95%	CI 95%
Years of schooling	−0.018	0.007	−0.011	−0.012	−0.017
	−0.052, 0.016	−0.027, 0.041	−0.039, 0.017	−0.044, 0.020	−0.040, 0.007
Age	0.000	0.020***	−0.005	0.006	0.012***
	−0.013, 0.013	0.007, 0.034	−0.017, 0.007	−0.006, 0.019	0.003, 0.021
Working [ref. not working]	−0.018	0.103	−0.006	0.058	−0.013
	−0.196, 0.161	−0.092, 0.298	−0.171, 0.159	−0.107, 0.224	−0.147, 0.121
Medium [ref. no overcrowding]	−0.033	0.103	−0.093	−0.027	−0.094
	−0.319, 0.254	−0.178, 0.385	−0.325, 0.139	−0.307, 0.253	−0.309, 0.121
High [ref. no overcrowding]	−0.124	0.593**	0.051	0.397*	−0.191
	−0.502, 0.254	0.088, 1.098	−0.258, 0.360	−0.034, 0.829	−0.462, 0.081
Critical [ref. no overcrowding]	−0.231	0.304	0.049	0.091	−0.247
	−0.758, 0.296	−0.216, 0.824	−0.518, 0.616	−0.464, 0.646	−0.913, 0.419
Married [ref. not married]	−0.092	−0.167*	−0.073	−0.245***	−0.057
	−0.250, 0.066	−0.342, 0.008	−0.217, 0.070	−0.400, −0.089	−0.177, 0.064
Income per−capita (log)	0.018	−0.018	−0.023	−0.003	−0.019
	−0.026, 0.063	−0.049, 0.013	−0.068, 0.023	−0.042, 0.036	−0.046, 0.008
Home IRT Std	0.067	0.006	−0.094**	−0.037	0
	−0.020, 0.153	−0.084, 0.096	−0.171, −0.017	−0.119, 0.046	−0.072, 0.072
Days of lockdown	0.002	0.003*	−0.001	0.001	0.001
	−0.001, 0.005	−0.000, 0.006	−0.004, 0.001	−0.002, 0.004	−0.001, 0.003
Partially complete lockdown [ref. failed to complete]	0.279*	0.324*	0.061	0.324**	0.041
	−0.029, 0.587	−0.053, 0.701	−0.249, 0.372	0.014, 0.634	−0.176, 0.258
Fully complete lockdown [ref. failed to complete]	0.315**	0.287	0.075	0.305**	−0.047
	0.026, 0.605	−0.063, 0.637	−0.217, 0.366	0.015, 0.594	−0.239, 0.145
Income decrease due to Covid [ref. no decrease]	−0.06	0.098	−0.011	−0.002	−0.002
	−0.225, 0.105	−0.089, 0.284	−0.170, 0.148	−0.164, 0.159	−0.127, 0.122
Received help due to Covid [ref. no help]	0.239	0.339*	−0.094	0.309**	0.044
	−0.059, 0.536	−0.001, 0.679	−0.388, 0.201	0.032, 0.586	−0.176, 0.265
Withdraw of retirement money [ref. no retirement]	0.167	0.353**	0.082	0.171	0.124
	−0.072, 0.406	0.067, 0.638	−0.129, 0.294	−0.051, 0.393	−0.054, 0.301
Change of housing [ref. no change]	−0.011	0.195*	0.311***	0.184*	0.146*
	−0.228, 0.206	−0.029, 0.418	0.138, 0.485	−0.012, 0.380	−0.007, 0.298
Family member with Covid [ref. no Covid]	0.087	−0.029	0.089	0.048	0.082
	−0.080, 0.255	−0.213, 0.154	−0.061, 0.239	−0.113, 0.210	−0.045, 0.210
Grief due to covid [ref. no grief]	0.228**	−0.013	0.014	−0.09	−0.051
	0.043, 0.413	−0.223, 0.197	−0.168, 0.197	−0.270, 0.090	−0.193, 0.091
CES-D IRT Std W1	0.449***				
	0.366, 0.533				
PSI-PD IRT Std W1		0.490***			
		0.398, 0.583			
PSI-DC IRT Std W1			0.300***		
			0.219, 0.380		
PSI-CDI IRT Std W1				0.225***	
				0.147, 0.302	
PACOTIS IRT Std W1					0.354***
					0.284, 0.423
Constant	−0.696	−2.189***	1.224***	−0.485	−0.029
	−1.621, 0.228	−3.089, −1.289	0.417, 2.032	−1.377, 0.406	−0.646, 0.588
Observations	679	679	679	679	679
R-squared	0.200	0.208	0.142	0.100	0.207

***p < 0.01. **p < 0.05, *p < 0.1.

Omitted/reference categories are mentioned following the ref—abbreviation term.

**TABLE 5:** Linear regression models foreign-born mothers (Mil Primeros Días, Santiago, Chile, 2019–2020).

	CES-D	PSI-PD	PSI-DC	PSI-CDI	PACOTIS
	β	Β	β	β	β
	CI 95%	CI 95%	CI 95%	CI 95%	CI 95%
Years of schooling	−0.054*	−0.085**	−0.044	−0.032	−0.050**
	[−0.112, 0.003]	−0.152, −0.017	−0.097, 0.010	−0.092, 0.028	−0.097, −0.003
Age	0.027*	0.017	0.012	0.003	0.015
	−0.005,0.058	−0.009, 0.042	−0.016, 0.041	−0.025, 0.031	−0.011, 0.041
Working [ref. not working]	−0.235	−0.296	0.004	0.053	0.165
	−0.699, 0.230	−0.746, 0.154	−0.359, 0.368	−0.293, 0.398	−0.117, 0.448
Medium [ref. no overcrowding]	0.348	0.115	0.228	0.038	−0.035
	−0.108, 0.805	−0.327, 0.558	−0.153, 0.610	−0.404, 0.479	−0.360, 0.291
High [ref. no overcrowding]	−0.158	0.079	−0.102	0.068	−0.200
	−0.727, 0.411	−0.290, 0.447	−0.570, 0.366	−0.328, 0.463	−0.673, 0.274
Critical [ref. no overcrowding]	−0.173	−0.235	−0.447	−0.455	−0.179
	−0.691, 0.345	−1.104, 0.635	−1.313, 0.418	−1.196, 0.285	−0.652, 0.294
Married [ref. not married]	0.192	−0.126	0.339*	0.012	0.147
	−0.251, 0.635	−0.470, 0.217	−0.040, 0.719	−0.404, 0.428	−0.201, 0.494
Income per-capita (log)	−0.043	0.106**	−0.032	0.043	0.011
	−0.101, 0.016	0.018, 0.195	−0.096, 0.032	−0.080, 0.166	−0.037, 0.059
Home IRT Std	−0.103	−0.055	−0.201*	−0.087	−0.117
	−0.297, 0.090	−0.265, 0.155	−0.429, 0.026	−0.282, 0.107	−0.285, 0.051
Days of lockdown	0.000	0.000	−0.002	0.005	−0.001
	−0.009, 0.009	−0.008, 0.007	−0.010, 0.006	−0.004, 0.013	−0.007, 0.006
Partially complete lockdown [ref. failed to complete]	−0.174	0.28	−0.087	−0.193	−0.128
	−0.966, 0.617	−0.711, 1.271	−0.624, 0.449	−0.861,0.475	−0.557,0.300
Fully complete lockdown [ref. failed to complete]	−0.041	0.557	−0.046	−0.055	−0.336
	−0.784, 0.703	−0.404, 1.518	−0.439, 0.346	−0.708, 0.598	−0.737, 0.065
Income decrease due to Covid [ref. no decrease]	0.337*	0.181	0.216	0.207	0.142
	−0.053, 0.727	−0.201, 0.563	−0.295, 0.726	−0.211, 0.625	−0.165, 0.448
Received help due to Covid [ref. no help]	−0.381*	0.177	0.027	−0.095	−0.089
	−0.793,0.031	−0.222,0.575	−0.415, 0.468	−0.531, 0.341	−0.437, 0.259
Withdraw of retirement money [ref. no retirement]	0.001	−0.07	−0.271	0.246	−0.200
	−0.372, 0.373	−0.493, 0.353	−0.632, 0.091	−0.157, 0.648	−0.496, 0.097
Change of housing [ref. no change]	0.327	0.074	−0.051	0.122	−0.075
	−0.064, 0.717	−0.301, 0.449	−0.382, 0.280	−0.218, 0.462	−0.316, 0.167
Family member with Covid [ref. no Covid]	0.154	0.105	0.330*	−0.046	0.218
	−0.232, 0.540	−0.278, 0.488	−0.045,−0.706	−0.394, 0.302	−0.074, 0.509
Grief due to covid [ref. no grief]	−0.033	0.011	−0.236	0.242	−0.155
	−0.470,0.404	−0.392,0.414	−0.640,0.167	−0.147,0.630	−0.504,0.195
CES-D IRT Std W1	0.371***				
	0.209, 0.533				
PSI-PD IRT Std W1		0.298***			
		0.155, 0.442			
PSI-DC IRT Std W1			0.328***		
			0.140, 0.517		
PSI-CDI IRT Std W1				0.206**	
				0.041, 0.372	
PACOTIS IRT Std W1					0.336***
					0.166, 0.505
Constant	0.414	−1.327	0.998	−0.64	0.556
	−1.603, 2.431	−3.501, 0.847	−0.664, 2.660	−2.867, 1.586	−0.941, 2.052
Observations	149	149	149	149	149
R-squared	0.254	0.236	0.238	0.142	0.293

***p < 0.01. **p < 0.05, *p < 0.1.

Omitted/reference categories are mentioned following the ref—abbreviation term.

Table 3 depicts coefficients for the whole sample. Results showed that foreign-born mothers have statistically significant higher scores—worse indicators—for parental distress, child dysfunctional interaction, and the hostility reactive dimension than native-born mothers. For PSI-PD and PSI-CDI, foreign-born mothers have 0.288 and 0.217 standard deviations, more on average than native mothers. The last column of the table shows results for the hostile dimension; foreign-born mothers scored 0.165 standard deviations more than native-born mothers, on average. Regarding covariates, years of schooling have a negative and statistically significant relation only with hostility, which means that more educated mothers have lower scores in this outcome. Mother’s age has a positive and statistically significant association with parental distress and hostility. As a categorical variable, overcrowding positively correlates with the test score only for parental distress and child dysfunctional interaction dimensions. Still, this result was statistically significant for the third category, high overcrowding, using as reference the non-overcrowded households. For the same two outcomes, being married appears to negatively correlated to the scores, meaning that married mothers experience less stress. The HOME indicator negatively correlates with the difficult child dimension, and its magnitude is 0.105 standard deviations.

On average, mothers who complied with lockdowns (partially and fully) got higher scores in two stress dimensions (parental distress and child dysfunctional interaction) and depressive symptoms than those who did not comply. Additionally, those mothers who received help from the government had 0.260 and 0.215 standard deviations more in parental distress and child dysfunctional interaction, respectively, compared to those that did not receive support. The same is true for those who withdrew retirement money; they have 0.274 and 0.213 standard deviations higher on parental distress and dysfunctional child interaction than mothers that did not withdraw their pension money. Change in housing is also associated with higher scores for difficult child, child dysfunctional interaction, and hostility—coefficients which were 0.227, 0.163, and 0.115-, respectively. And grief due to COVID had a positive and statistically significant association with depressive symptoms. Table 3 highlights the importance of controlling for the instrument’s previous score, showing a positive and statistically significant association at a 99% confidence level for the five outcomes analyzed in this study.

As we mentioned above, Tables 4, 5 show the coefficients and confidence intervals (95%) for the estimations separated by group: native-born and foreign-born mothers, respectively. For native mothers (see Table 4), years of schooling do not appear to be an essential determinant of depressive symptoms, stress, and hostility after 8 months of the pandemic. However, the mother’s age is important for one of the PSI dimensions and hostility towards children. An additional year of the mother’s age worsens the outcomes. Being married acts as a protective factor against stress, parental distress and child dysfunctional interaction, reducing these outcomes, and showing that married mothers have lower stress levels. Compliance with lockdown increases depressive symptoms and two of the dimensions of stress. For native mothers, receiving governmental aid and withdrawing retirement money are determinants of more stress. If the family experienced a housing change during the period, that also increased levels of stress and hostility. For foreign-born mothers (see Table 5), years of schooling are positively associated with depressive symptoms, stress, and hostility. After 8 months of the pandemic, more educated mothers have lower depressive symptoms, less stress, and less hostility. Except for the lagged value from the test score, all of the other covariates do not seem relevant to explaining this group’s mental health outcomes.

## Discussion

Our findings suggest heterogeneity in the association between the COVID-19 pandemic and maternal depressive symptoms, maternal stress, and maternal practices between foreign-born and native-born mothers of young children (24–30 months old) in Chile. This study shows evidence that the COVID-19 pandemic can have heterogeneous effects on depressive symptoms and stress levels, affecting foreign-born mothers to a greater extent. Foreign-born mothers have worse parental distress, child dysfunctional interaction, and hostile-reactive parental behavior than native-born. More specifically, migrant mothers scored 0.288 standard deviations more in the parental distress subscale of the Parental Stress Index, similar to the result for the parent-child dysfunctional interaction subscale. They exceeded their native counterparts, obtaining 0.217 standard deviations more on average. Foreign-born mothers had scores 0.165 standard deviations higher on average.

This study contributes to the emerging literature that found evidence that the COVID-19 pandemic has had negative consequences on depressive symptoms and stress levels of the population, adding an important unexplored evidence about foreign-born population. These consequences are worse for mothers and vulnerable and marginalized groups (Moya et al., 2021). Furthermore, the analysis focused on native-born and foreign-born mothers in Chile with children aged between 24 and 30 months old. Our results showed that foreign-born mothers’ depressive symptoms, stress, and practices have been more affected than native mothers. Inflows of migration are increasing in Chile. In that frame, relevant topics for future research are the conditions in which this population lives and how pandemics will affect them in the long term. In addition, our data showed significant differences in access to social protection from the government, withdrawal of money from retirement funds, and housing changes between native and foreign-born mothers. To some extent, that could explain the differences in mental health that we have found between these two groups.

Maternal mental health issues are associated with short-term and long-term risks for the mother’s health and function and affect their children’s physical, cognitive and psychological development [26]. Our results are keys for implementing targeted public policy initiatives to prevent long-term effects on mental health, and mitigate or even negative consequences for children during an essential stage of their development.

In actual context, identifying the most vulnerable populations is crucial to delivering targeted support and ensuring the efficient use of limited resources. Supporting mothers with young children has a double effect. It directly helps women enhance their mental health, helping them overcome the COVID-19 pandemic, and, indirectly, reduces the potential impact on children. Besides providing the most affected mothers with psychological and monetary support, parenting programs should be implemented to enhance parent-child relationships and mitigate the long-term effects that this pandemic can have on children. Evidence-based parenting programs have been shown to positively affect children in other low-middle income countries [27, 28].

It is worth mentioning that our study has some limitations. First, the *Mil Primeros Días* sample does not represent the whole country. Mothers selected are from the Metropolitan Region and only from users of the public health system (it does not include 20% of the population that uses the private health system). Therefore, our results do not include the wealthiest people in the region. Moreover, even if we have longitudinal data and can control for the instrument’s score from the period before the pandemic, we cannot distinguish a causal effect.

Further research should be conducted on other possible vulnerable populations in Chile and elsewhere. Additionally, it is necessary to continue studying mental health in people affected more severely by the pandemic to maintain awareness about the mid-term and long-term effects that COVID-19 can have. Finally, we propose that central and local governments support and guide families and individuals to overcome the pandemic and minimize negative consequences. We believe that it is crucial to investigate psychosocial resilience factors at individual, family, and community levels to help deal with future health crises, which is especially relevant for the most vulnerable such as migrant mothers of young children.
